# Systemic Autoimmunity in TAM Triple Knockout Mice Causes Inflammatory Brain Damage and Cell Death

**DOI:** 10.1371/journal.pone.0064812

**Published:** 2013-06-20

**Authors:** Qiutang Li, Qingjun Lu, Huayi Lu, Shifu Tian, Qingxian Lu

**Affiliations:** 1 Department of Ophthalmology and Visual Sciences, University of Louisville, Louisville, Kentucky, United States of America; 2 Department of Anatomical Sciences and Neurobiology, University of Louisville, Louisville, Kentucky, United States of America; 3 The James Graham Brown Cancer Center, University of Louisville, Louisville, Kentucky, United States of America; 4 School of Basic Medicine and Beijing Tong-Ren Hospital, Beijing Ophthalmology and Visual Science Key Laboratory, Capital Medical University, Beijing, China; University of Illinois at Chicago, United States of America

## Abstract

The Tyro3, Axl and Mertk (TAM) triply knockout (TKO) mice exhibit systemic autoimmune diseases, with characteristics of increased proinflammatory cytokine production, autoantibody deposition and autoreactive lymphocyte infiltration into a variety of tissues. Here we show that TKO mice produce high level of serum TNF-α and specific autoantibodies deposited onto brain blood vessels. The brain-blood barrier (BBB) in mutant brains exhibited increased permeability for Evans blue and fluorescent-dextran, suggesting a breakdown of the BBB in the mutant brains. Impaired BBB integrity facilitated autoreactive T cells infiltrating into all regions of the mutant brains. Brain autoimmune disorder caused accumulation of the ubiquitin-reactive aggregates in the mutant hippocampus, and early formation of autofluorescent lipofuscins in the neurons throughout the entire brains. Chronic neuroinflammation caused damage of the hippocampal mossy fibers and neuronal apoptotic death. This study shows that chronic systemic inflammation and autoimmune disorders in the TKO mice cause neuronal damage and death.

## Introduction

Tyro3, Axl and Mertk belong to a structurally and functionally closely-related TAM family of receptor tyrosine kinases. Both Gas6 and Protein S serve as ligands for this family of receptors. Mice lacking all three members of the receptors developed severe autoimmune diseases [1], [2], largely due to unrestricted activation and cytokine receptor signaling on the antigen-presenting cells [3], [4], and eventually develop spontaneous autoimmunity with characteristics of splenomegaly, glomerulonephritis, higher frequency of activated APCs and lymphocytes, production of a wide spectrum of autoantibodies reacting with nuclear antigens, double-stranded (ds) DNA and phospholipids, exhibit a typical manifestation of human systemic lupus erythematosus diseases (SLE)-like [1], [5]. *Tyro3^−/−^Axl^−/−^Mertk^−/−^* triple (TKO) or *Axl^−/−^Mertk^−/−^* double (AM DKO) knockout mice have recently been shown to spontaneously develop autoimmunity against retinal specific autoantigens and are more vulnerable to retinal antigen immunization with a dominant Th1 effort response [6], [7].

Although all three members of receptors are expressed in brain, Tyro3 is strongest expressed in cortical and hippocampal neurons [8], [9], however, Tyro3 single knockout mouse does not show noticeable neuronal pathology [1], [10]. In brain, Tyro3 is also expressed by microvessel endothelial and vascular smooth muscle cells, and functions as trophic factor to support cell proliferation, migration and survival. Loss of this receptor, impaired brain microvessel integrity, endothelial cell adhesion, eventually leading to brain-blood barrier (BBB) disrupt and leakage [11]. On the other hand, the ligand protein S protects BBB integrity from oxygen/glucose deprivation-induced BBB breakdown through Tyro3 mediated vasculoprotection [11]. Mice without protein S develop lethal embryonic coagulopathy, ischemic/thrombotic injuries, vascular dysgenesis, and BBB disruption with intracerebral hemorrhages [12], [13]. In addition, Axl and its ligand Gas6 expressed in vascular smooth muscle and endothelial cells were upregulated during vascular injury, likely to support cell migration and suppress apoptosis [14]–[16].

Brain is normally protected from insulting attack from peripheral immune system by BBB that is formed by capillary endothelial cells lining brain microvessels. When the BBB is disrupted, large molecules and blood cells can invade into brain and spinal cord, leading to central nervous system (CNS) damage. Systemic autoimmunity and increased proinflammatory cytokines, such as TNF-α, increase the permeability of brain microvascular endothelial cells [17]–[22]. Studies on lupus patients or several spontaneous mouse models of lupus, including the (NZBxNZW) F1 (BWF1) hybrid, BXSB and MRL-lpr mice show that neuroinflammation is a major contributing factor to CNS pathology and dysfunctional behavior; and the autoantibodies and self-reactive T cells are able to penetrate into neuronal tissues causing neurodegeneration and CNS atrophy [23]–[25]. Systemic lupus erythematosus patients, usually containing low serum protein S in their blood [26], are frequently accompanied by neuropsychiatric and cognitive deficits, and those neuropsychiatric disorders were largely caused by progressive neuronal death and hippocampal damage.

Our previous studies showed that the brain blood endothelial cells were obviously activated based on the increased expression of ICAM on the endothelial surface of cerebral microvessels [1], which plays important role in facilitating the movement of immune cells into the brain during inflammatory responses, such as multiple sclerosis and experimental autoimmune encephalomyelitis (EAE) [27], [28]. In the present study, we aim to further evaluate autoimmune damage to TAM TKO mouse brain. Our results showed that antibody deposition and autoreactive T cells accumulation occurred in TKO brains; and the brain blood vessel permeability was dramatically increased in the mutants. As a consequence, the mutant brains exhibited glial activation, ubiquitinized protein aggregation, hippocampal damage and programmed cells death. This study provides a new target for preventing inflammatory damage to central nerve system.

## Materials and Methods

### Animal and Ethics Statement

The TAM gene knockout mice, which were created on the C57BL/6 and 129 mixed background [10], have been backcrossed to the wild-type pure C57BL/6 background for at least 11 generations in our laboratory. All animals were housed in a pathogen-free facility and were handled according to the regulations of the Institutional Animal Care and Use Committee (IACUC), No.10131.

### Immunofluorescence

For immunostaining, the frozen brain sections were prepared and incubated in blocking solution (10 mM Tris-buffered saline (TBS) containing 0.1% Triton X-100, 5% normal serum prepared from the secondary antibody host species) for 1 hr at room temperature, and by incubation with primary antibodies in the same buffer for 24 to 48 h at 4°C. After washing with TBS plus 0.1% Triton X-100 for 4 times with 5 min each, the sections were further incubated with the secondary antibodies in the blocking solution for 1–2 h at room temperature in a dark, humidified chamber. Sections were then washed with TBS plus 0.1% Triton X-100 and coverslipped in Vectashield containing DAPI for nuclear staining (Vector Laboratories, Burlingame, CA). The primary antibodies were applied in the following concentrations: Goat anti mouse GFAP (1∶200, Santa Cruz), rabbit anti-calbindin-28 (1∶300, EMD Millipore), Mouse anti-ubiquitin (1∶100, RnD), rabbit anti-CD3 (1∶100, DakoCytomation). Secondary antibodies were purchased from Jackson ImmunoResearch laboratory (West Grove, PA) and used as follows: Cy3 or DyLight-488 conjugated donkey anti-mouse (1∶250), Cy-3 or DyLight-488 conjugated donkey anti-rabbit antibodies (1∶250); DyLight 488 conjugated donkey anti-goat (1∶250).

### Timm Staining

After deep anesthesia, the mice were intracardially perfused with 1×PBS for 1 min and followed by 1.2% sodium sulfide in 0.1 M phosphate buffer, pH 7.4, for 1 min, then with 3% glutaraldehyde in phosphate buffer, pH 7.4 for 3 min, and next followed by 7 min with the sodium sulfide solution. After dissection, the brains were removed from the skull and post immerse-fixed in 3% glutaraldehyde for 1 h prior to sectioning at 50 µm in 0.1 M Tris (hydroxymethylaminomethane) buffer, pH 7.6, using a Vibratome (Leica). Five brains from each group were cut to obtain the coronal hippocampal sections. After mounted the sections were rinsed briefly in a jar with 1×PBS and covered with the physical developer (60 mL of 50% gum Arabic, 10 mL of 2 M citrate buffer, pH 3.7, 30 mL of 5.67% hybroquinone, and 0.5 mL of 17% silver nitrate) in dark at 26°C for 75 min, and then at 60°C for 20 min. After rinsed in dH_2_O, the sections were counterstained in hematoxylin for 5 min, dehydrate and cleaned with 2× xylene and mounted in permamont.

### TUNEL (Terminal Deoxynucleotidyl Transferase-mediated dUTP Nick End Labeling) Histochemistry

Paraffin-embedded brain tissues were coronally sectioned at 20 µm, followed by routine histological deparaffinization and dehydration procedures. The dehydrated sections were digested with 20 µg/mL of freshly prepared proteinase K in 10 mM Tris-HCl, pH 7.4 buffer for 15 min at room temperature. After wash with 1×PBS for 4 times, the brain sections were covered with 50 µl of TUNEL reaction (Roche Diagnostics, Indianapolis, IN) and incubated at 37°C for 1 h in a dark and humidified chamber. After TUNEL staining, sections were coverslipped with Vectashield containing DAPI for nuclear staining (Vector Laboratories, Burlingame, CA). Images were taken using a Leica confocal microscope with excitation at 488 nm and emission at 545 nm.

### Brain-Blood-Barrier Permeability Assays

For Evans blue dye (Sigma-Aldrich) as tracers to assess the brain vascular permeability, both TKO (n = 6) and wild type (n = 6) female mice at ages of 8–10 months were intravenously injected via the tail vein with either Evans blue (30 mg per 1 kg body weight in a 6 mg/mL solution) for 30 min prior to euthanasia. At the end of assays, the mice under deep anesthesia, the brains were rapidly removed and embedded in O.C.T medium. The frozen sections were cut at 30 microns and fixed quickly for 1 min in the cold acetone. After air dried at room temperature, the sections were dipped into xylene and mounted with glass cover-slip. The EB inside the brain microvessels and this penetrating into parenchyma were observed under fluorescent microscope using a red fluorescent filter with an excitation wavelength at 540 nm and emission peak at 680 nm.

For fluorescent quantification of Evans blue retaining in the brains, the brain tissues obtained from PBS perfused WT (n = 5) and TKO (n = 5) mice were weighed and homogenized in formamide, followed by incubation at 56°C for 30 min, and centrifugation for 15 min at maximal speed in a Beckman Coulter microfuge-18 centrifuge. The Evans Blue retained in the supernatant was measured by absorbance at the optical density (OD) of 600 nm on a fluorescent spectrophotometer (Molecular Devices, CA). Absorbance was normalized to the sample weight.

For FITC-Dextran assessment of BBB leakage, mice under anesthesia were intravenously injected via the tail vein with fluorescein isothiocyanate (FITC)-dextran, 70 kDa (Invitrogen) for 20 min, which allows dextran to circulate throughout the vasculature. After 20 min, the mice were transcardially perfused with 1×PBS containing 1 unit/mL of heparin for 15 min, followed by 60 mL of 4% paraformaldehyde. The brain was extracted from the skull and post-fixed in paraformaldehyde overnight followed by cryoprotection in 20% sucrose overnight. The brain tissue was frozen-embedded in O.C.T, and sectioned into 30 µm-thickness in a coronal orientation using a cryostat. The sections were counter-stained with DAPI and imaged with a deep-cooled CCD imaging system and the images were obtained and analyzed using Carl Zeiss imaging systems software version 4.8.2.

### Isolation and Culture of Mouse Brain Endothelial Cells

Brain vascular endothelial cells were isolated from WT or *Tyro3^−/−^Axl^−/−^* double knockout mice according to published protocols [22]. Briefly, under deep anesthesia, the mouse brains were dissected and rinsed with sterile RPMI-1640 medium containing 1× penicillin and streptomycin (Life Technologies). Meninges and large blood vessels were removed carefully, and the remaining brain tissue was cut into small pieces and homogenized in RPMI-1640 plus 2% FBS using a loose Dounce homogenizer. An equal volume of 30% dextran solution was then added to homogenates to make a 15% dextran density gradient and centrifuged at 3000×g for 25 min. The tissue pellet containing microvessels was resuspended in 3 mL DMEM (Life Technologies) containing DNase-I, 10 mg/mL and collagenase IV, 2 mg/mL at 37°C in a shaking water bath for 30 min. After centrifugation, the cell pellets were washed once in medium and plated onto rat-tail collagen-coated dishes (BD Biosciences) and cultured in complete M131 medium (Life Technologies) supplemented with 1× penicillin/streptomycin, 1× Glutamax-I and 1× Microvascular Growth Supplement (MVGS) reagents (Life Technologies). Twenty-four hours after plating, unattached cells and cell debris were removed, and fresh culture medium was replaced with every 2 days until confluent.

### FITC-Dextran Permeability

The permeability of the brain blood vessel endothelial cells to Fluorescein isothiocyanate (FITC)-dextran (70 kDa, Sigma-Aldrich) was assessed in the primary cultured endothelial monolayers. Seven days after isolation, the confluent cells were trypsinized to make single cell suspension, and 10^4^ cells in 0.1 mL complete M131 medium were plated into each upper chamber in a 24-well transwell plate (0.4 µm, Corning Costar, Rochester, NY, USA), and incubated for 4 more days to form confluent endothelial monolayer. Tissue culture medium was then removed and replaced with fresh warm medium containing 70 kDa FITC-dextran (1 mg/mL) to the upper chamber of each well and incubated at 37°C for 120 minutes. The lower chamber containing 0.8 mL phenol-red free DMEM was sampled (20 µL) at indicated timepoints. Each sample was diluted 1∶10 in 1×PBS; and the fluorescence intensity of each diluted sample was determined (excitation and emission were 485 nm and 528 nm, respectively), using a fluorescence multiwell plate reader (Synergy-2 multi-mode microplate reader, Bio-Tek Instruments, Inc., Vinooski, VT). The amount of FITC–dextran diffused to the bottom chamber was determined and expressed as arbitrary fluorescent intensity. The data were obtained by plotting fluorescent intensity against the time of FITC-dextran fluxes.

### RNA Isolation, cDNA Synthesis, and Real-time Quantitative PCR

Total RNA from lymph nodes was extracted using TRIzol reagent, following the manufacturer’s instruction (Invitrogen, San Diego, CA). 2 µg of total RNA from each sample was treated with DNase I to remove traces of genomic DNA, and then was reverse transcribed into first-strand cDNA using a qScript cDNA SuperMix kit (Quanta Biosciences, Gaithersburg, MD) for real-time quantitative PCR (qPCR) analysis. Real-time qPCR was performed in a SYBR green-based PCR reaction mixture on a MX3005p system (Agilent Technologies, Santa Clara, CA), with a program of a 10-min initial hot-start activation of Taq polymerase at 95°C, followed by 40 cycles of amplification (95°C for 10 s, 56°C for 5 s, and 72°C for 10 s). After amplification, a melting curve was generated by holding the reaction at 65°C for 15 s, then heating to 95°C, with a ramp rate of 0.1°C/s. To obtain the melting temperature for each sample, the fluorescence signal was plotted against temperature. The comparative threshold cycle method normalized to β-actin was used to analyze relative changes in gene expression.

The oligonucleotides used for qPCR were 5′-TGGAGAGTGTGGATCCCAAGCAAT-3′ and 5′-TGTCCTGACCACTGTTGTTTCCCA-3′ for IL-1β, 5′-TGGCTAAGGACCAAGACCATCCAA-3′ and 5′-AACGCACTAGGTTTGCCGAGTAGA-3′ for IL-6, and 5′-GGCTGTATTCCCCTCCATCG-3′ and 5′-CCAGTTGGTAACAATGC-CATGT-3′ for β-actin.

### Peritoneal Macrophage Preparation, LPS Treatment and TNF-α ELISA Assay

Peritoneal macrophages were prepared from day 4 of 3% thioglycollate induced WT and TKO mice, detailed procedures was described previously [6]. Briefly, 3×10^6^ cells were plated into each well of a six-well plate. Nonadherent cells were removed 2 h later, and the remaining adherent cells were cultured in complete medium for 3 d, and then were treated with 100 ng/mL LPS for indicated time points before harvesting the culture medium for TNF-α quantification. TNF-α level in the mouse serum or macrophage culture medium were measured using the TNF-α ELISA kits and following the manufacturer’s instruction (eBiosciences, CA).

### Brain Mononuclear Cell Preparation and Flow Cytometric Analysis

The brains were dissected from the WT and TKO mice that had been anesthetized and perfused through the left ventricle with 1× HBSS. Each brain was homogenized in 3 mL of RPMI medium using a Dounce homogenizer. The homogenized tissue preparation was diluted with 70% Percoll in HBSS to make a final 30% of Percoll in the tissue suspension, which was then slowly placed on the top of the 70% Percoll in HBSS. This 70%–30% gradient was centrifuged at 500×g for 30 min at room temperature. After centrifugation, 3.0 mL of the 70%–30% interphase containing mononuclear cells was saved and washed twice with RPMI supplied with 2% FBS. After block of the Fc with anti-CD16/32 antibody, about 10^6^ cells in 50 µl staining buffer (PBS containing 3% BSA and 0.1% sodium azide) were incubated at 4°C for 30 min with FITC-conjugated anti-TCRα mAb (clone H57-597, eBiosciences) and analyzed on a four-color BD FACSCalibur (BD Biosciences), the data was processed with CellQuestPro 5.1.1 software (BD Biosciences).

### Mouse Serum Antibody Isotyping

Briefly, the serum was prepared from the mice with indicated genotypes and diluted at 1∶5000 in 1×PBS before coated onto ELISA plates. The subclasses of serum antibody were measured using mouse monoclonal antibody isotyping kit following manufacturer’s instruction (Sigma-Aldrich).

### Statistics

Data are presented as means ± SD. The unpaired *t* test was used for two group comparisons and ANOVA Tukey’s multiple comparison tests were used for analysis of differences in three or more groups. Statistically significant levels are indicted as follows: *P<0.05, **P<0.001.

## Results

### Autoantibodies Deposit onto Brain Microvessels in the TAM TKO Mice

**Figure 1:**
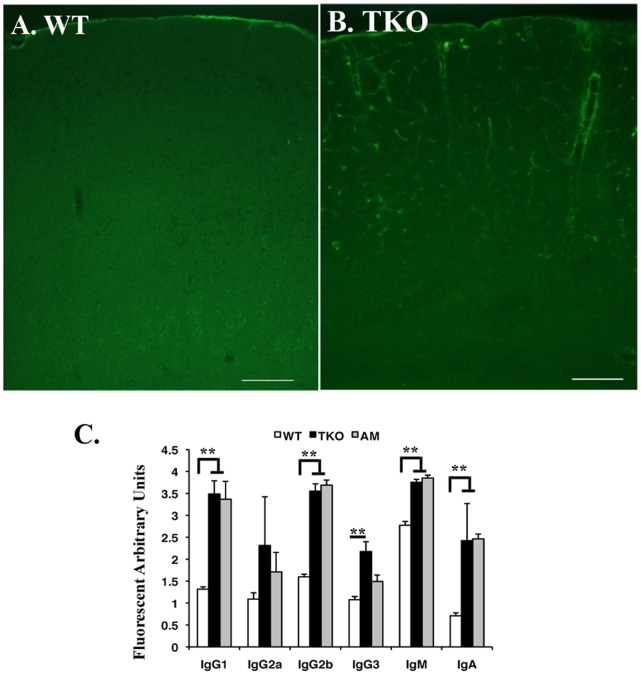
Autoantibodies are deposited on the microvessels of the TKO brain. Both WT (A) and TKO (B) mice at age of 8 weeks were deep anesthetized by 2.5% Avertin and the blood vessels were flushed through transcardial perfusion using hepatinized 1×PBS for 5 min followed by 8 min of 4% PFA perfusion fixation. After dissection, the brain tissues were postfixed in 4% PFA at 4°C for overnight. Coronal sections at the middle of cortex were cut on cryostat and stained with FITC labeled rabbit-anti-mouse IgG. The images were taken on fluorescent microscope. Bars, 200 µm. (C) Serum antibody isotyping was performed on the sera isolated from WT, TKO and *Axl^−/−^Mertk^−/−^* (AM), using mouse monoclonal antibody isotyping reagents following manufacturer’s instruction (Sigma-Aldrich). The data is expressed as mean±SD, n = 6. The statistics was performed by ANOVA Tukey’s multiple comparison tests using Prostat ver5.5 program **P<0.001.

Given that the mice lacking TAM receptors develop systemic autoimmunity spontaneously [1] and neuropsychiatric lupus (NP-SLE) patients are frequently accompanied by increased levels of serum autoantibodies reacting with brain specific antigens [29], we examined whether antibody deposition occurred in TAM TKO brains by immunohistochemistry with anti-mouse IgG antibody. Immunohistochemistry with anti-mouse IgG antibody clearly demonstrated massive IgG deposition along the endothelial surface of the TKO brain microvessels (Fig. 1B), which was in clear contrast to negligibly staining on the WT brain sections (Fig. 1A). Anti-IgM antibody demonstrated a similar immunostaining pattern (data not shown). Such antibody deposition in brain can be detected in TKO mice at age of 2 month old, by the age that the TKO mice showed enlarged spleens and lymph nodes [1]. Identification of immunoglobulin subclasses showed significant increased IgG1, IgG2b, IgM and IgA isotypes in the mutant sera (Fig. 1C), suggesting an isotype switch occurred in the mutants.

### TKO Mice Produced Increased TNF-α in Serum

**Figure 2:**
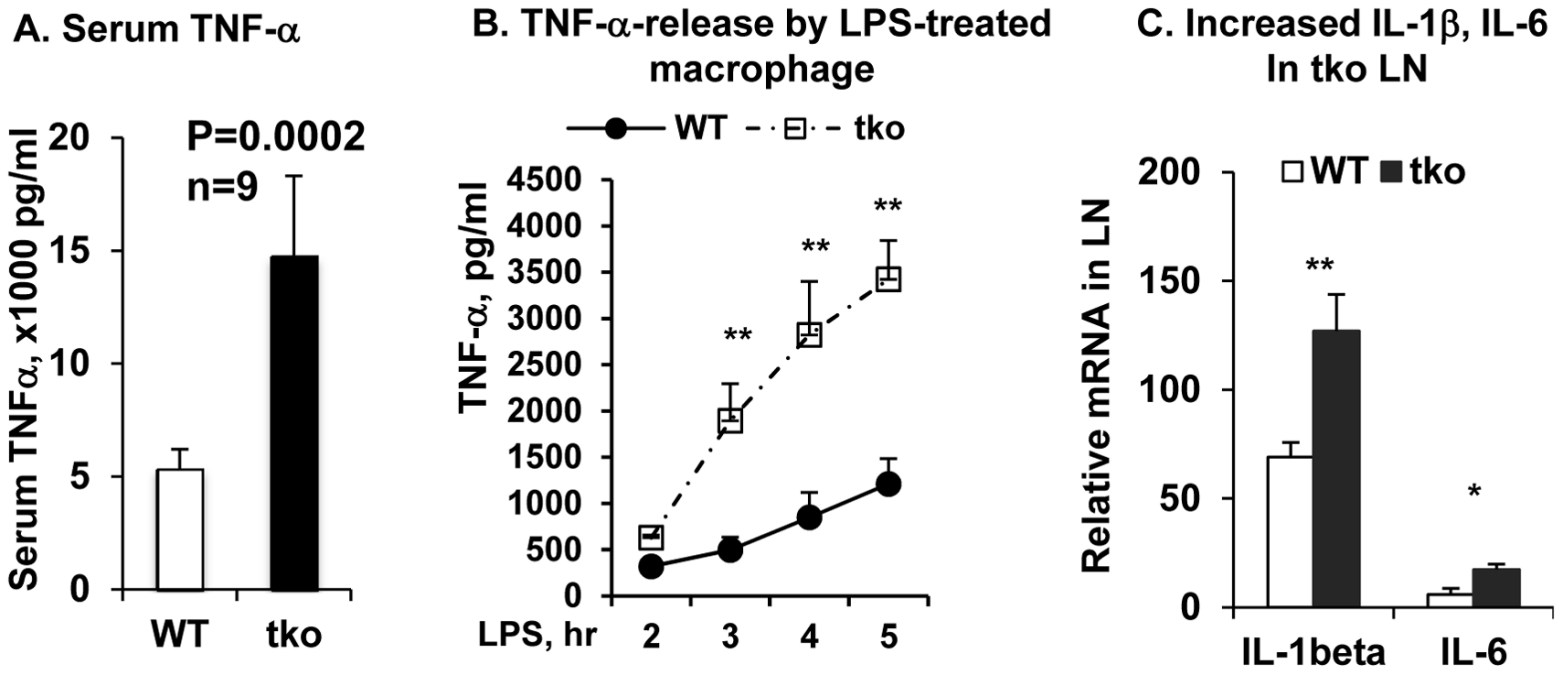
TKO mice produce increased proinflammatory cytokines. (A) TNF-α level in the WT and TKO mouse serum were measured by Ready-set-go ELISA kits (eBiosciences). Data are shown as means±SD, n = 9, p = 0.0002. (B) Thioglycollate-induced peritoneal macrophages (MФ) were treated with LPS for 2, 3, 4 and 5 hrs. TNF-α released into medium was measured as above. Data are shown as means ±SD for five wells per group in a single experiment and are representative of those in three experiments. N = 3, **P<0.001. (C) Real-time qPCR quantification of IL-1β and IL-6 mRNA in the lymph nodes. The total RNA was extracted by TRIzol and reversely transcribed using transcribed using qScript™ cDNA Supermix kit (Quanta Biosciences, MD). Real time qPCR was performed to measure the relative mRNA level of IL-1β and IL-6 genes in the WT (open) and TKO (solid) L.N. Data are shown as means ± SD, n = 3, *P<0.05, **P<0.002. Statistics was performed by the one-way ANOVA test using ProStat Ver 5.5.

In consistent with high levels of TNF-α in the LPS challenged Mertk single gene knockout mice [30], TKO mice exhibited even higher level of TNF-α in circulation (Fig. 2A). TKO mice showed increased total numbers of macrophage, DCs, NK and activated CD4^+^ T cells [1], [3], [6], [31], which may contribute to such increased TNF-α in circulation. While all those cells are capable, macrophage is a primary cell type to produce TNF-α. To test whether or not the high serum level of TNF-α in TKO mice is attributed to increased release of TNF-α by each individual cells in response to LPS stimulation, we cultured equal number of 3% Thioglycollate-induced peritoneal macrophages prepared from wild-type and TKO mice and treated with 100 ng/mL of LPS for 0, 1, 2, 3, 4 and 5 hr, and the TNF-α released into culture medium was measured by ELISA. TKO macrophage exhibited enhanced release of TNF-α upon LPS stimulation (Fig. 2B), suggesting that increased TNF-α in the TKO mouse serum was caused by hyperreactivity of the mutant cells, consistent with a negative regulatory role of TAM receptors on DCs and macrophages [3], [32]. Increased production of TNF-α by the TKO mice was further supported by the increased expression of other proinflammatory cytokines, such as IL-1β and IL-6 (Fig. 2C), both of which are under similar regulatory network during the inflammatory responses.

### Permeability in the TKO Brain Blood Vessels is Increased

**Figure 3:**
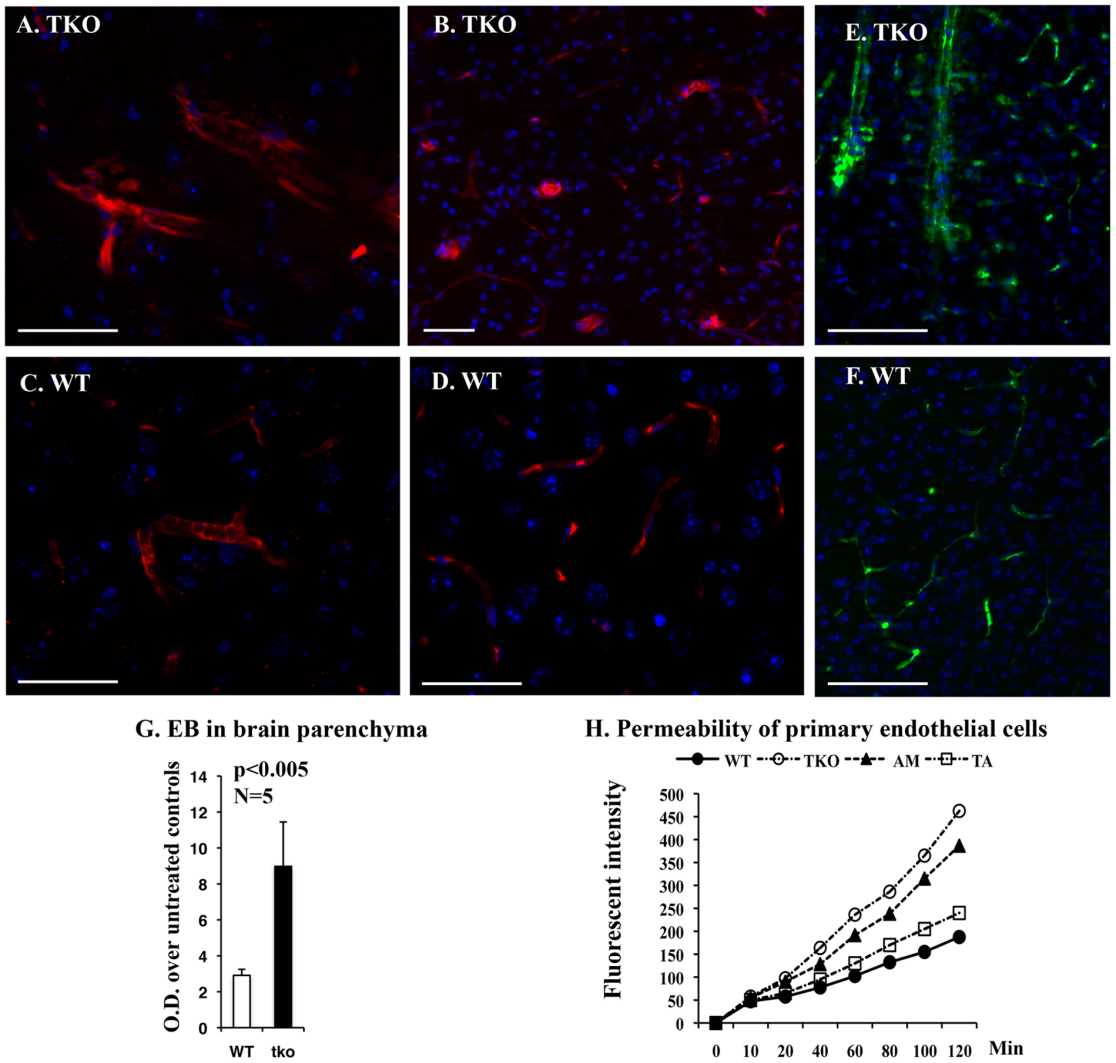
The BBB integrity is altered in TKO mice. (A–D) Mice at 6–8 months of age were *i.v*. injected with Evans blue for 30 min. Histological and image processes follow procedure described in Methods and Materials. Extravasation of Evans blue into parenchyma can be clearly detected in the TKO brains (A and B), in contrast to barely-detected leakage in the WT brain (C and D). (E, F) Mice at 6–8 months of age were *i.v*. injected with FITC-dextran (70 kDa) for 20 min prior to systemic transcardial perfusion of 1×PBS supplemented with 1 unit/mL of heparin. Brains were harvested, cryosectioned at 30 µm in thickness and stained with DAPI. Fluorescent images in (A–F) were observed using a Zeiss microscope equipped with Apotome and obtained with a deep-cooled CCD imaging system, and analyzed using Carl Zeiss imaging systems software version 4.8.2. Scale bars in (A–F) are 50 µm. (G) For fluorescent measurement of Evens blue retained in brains, the brain tissues obtained from WT and TKO mice that had been perfused with hepatinized PBS were weighed and homogenized in formamide, followed by incubation at 56°C for 30 min, and centrifugation for 15 min at maximal speed in a Beckman Coulter microfuge-18 centrifuge. The Evans blue retained in the supernatant was measured by absorbance at the optical density (OD) of 600 nm on a fluorescent spectrophotometer (Molecular Devices, CA). Absorbance was normalized to the sample weight. Data is expressed as mean ±SD, n = 5, p = 0.005. (H) The integrity of the brain blood vessel endothelial cells was measured using a transwell permeability assay system. The brain endothelial cells were isolated from WT, TKO, AM and TA mice at age of postnatal day 12 and cultured for 7 days in medium 131 supplemented with microvascular growth supplements (Life Technologies). Cell permeability assay was described in the Methods and Materials. The value at each time point in each genotype was the mean of three independent cultures, and each culture was pooled endothelial cells from at least four pups with same genotype.

TNF-α has been considered as an important inducer of enhanced blood-brain barrier (BBB) permeability [19]–[21], one of which mechanism is thought to upregulate intracellular adhesion molecule-1 (ICAM-1) [18] on the brain microvessel endothelial cells; and the ICAM-1 was previously found up-regulated in the TKO brain [1]. TAM receptors are expressed in blood vessel endothelial cells and participate in regulation of endothelial cells tight junction formation [11]. We therefore examined the BBB permeability for the large mass of molecules in the TKO mice. We first visualized the Evans blue infiltration into brain parenchyma under fluorescent microscope, the blood vessel leakage was clearly evident in the TKO brains (Fig. 3A and 3B) but not in the WT control brains (Fig. 3C and 3D). In addition, extravasation of FITC-dextran (70 kDa) into parenchymal tissue after 20 min *i.v.* injection was clearly evident around the large size of blood vessels in the TKO but not in the WT brains (Fig. 3E versus 3F). Interestingly, this increased brain blood vessel permeability was mainly noticed in the large and mid-size blood vessels, but not in the microvessels (Fig. 3B versus 3D). The BBB permeability for Evans blue was further evaluated by fluorescent measurement of the brain parenchyma-retained Evans blue after 30 min of tail veil injection into either WT or TKO mice with the ages between 8–10 month old. The TKO brains retained approximately three times as much as the WT brains (Fig. 3G). Increased permeability for large mass of proteins in the TKO mice may be resulted from chronic inflammation or from the impaired endothelial integrity, we therefore tested the permeability of monolayer endothelial cells prepared from *Tyro3^−/−^Axl^−/−^* (TA), *Axl^−/−^Mertk^−/−^* (AM), and *Tyro3^−/−^Axl^−/−^Mertk^−/−^* (TKO) compound mice. The integrity of the endothelial cell barriers in the cultured double and triple gene knockout cells was interrupted, with severity of TKO>AM>TA, as compared to the WT controls (Fig. 3H), suggesting that TAM receptors play roles in keeping the integrity of the blood vessel endothelial cells.

### T cells Infiltration into TKO Brains

**Figure 4:**
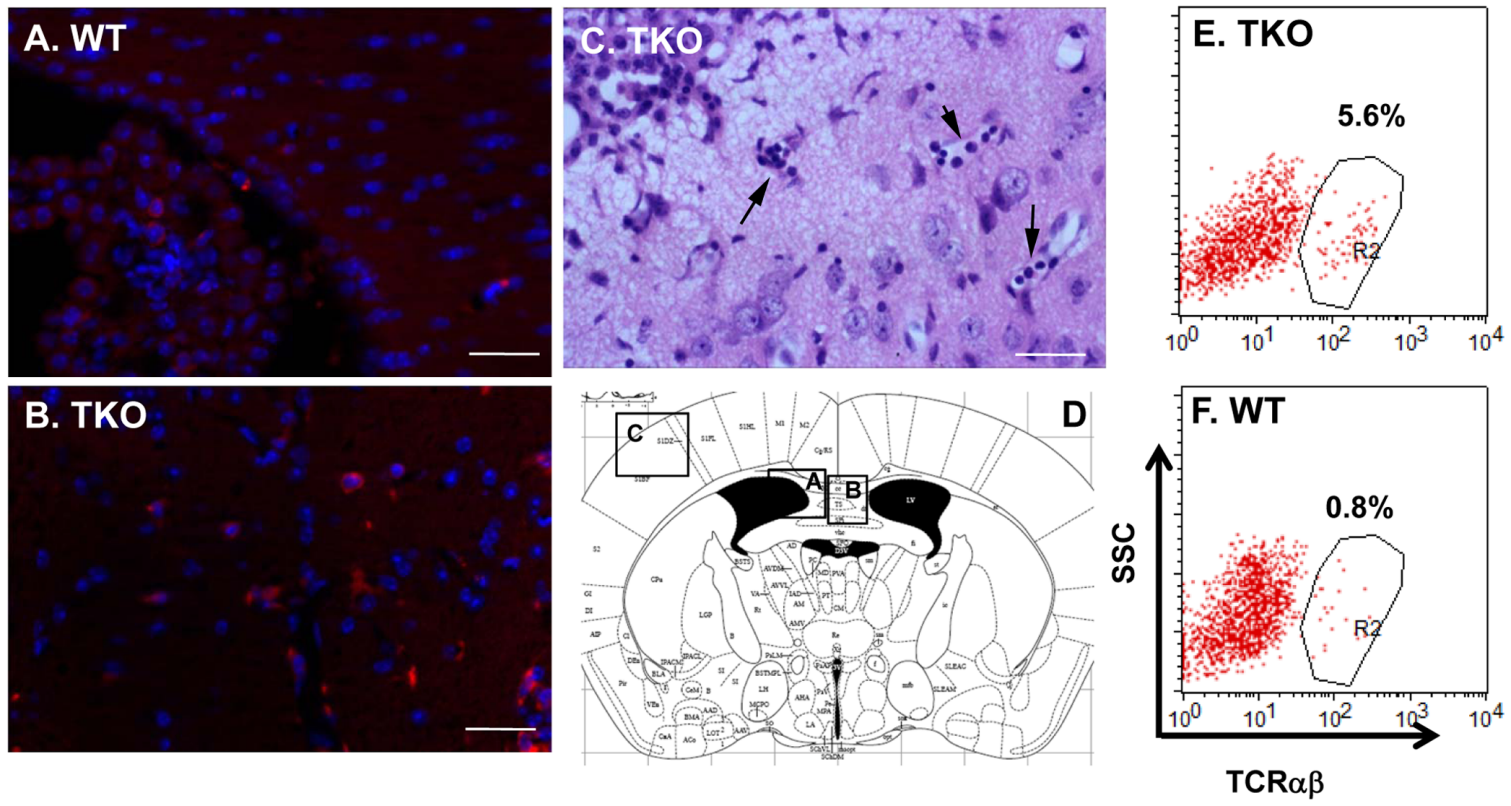
T lymphocytes invade into TKO brain. (A, B) The WT (A) and TKO (B) mice at 7 weeks of age were prepared for brain sections, which were immunostained with anti-CD3 antibody to show T cell infiltration. (C) The brain section from the TKO mouse at age of 10 month old was stained with hematoxylin and eosin (H&E). Scale bars, 50 µm. (D) Brain diagram shows the regions of figures A–C. (E, F) Flow cytometric analysis of infiltrated TCRαβ-positive cells in TKO brains. Cell preparation and flow cytometry procedures were described in the Methods and Materials. There are increased TCRαβ-positive cells in the TKO brain (E, 5.6% of leukocytes) than the WT brain (F, 0.8% of leukocytes). This is one representative for each genotype, n = 3.

Disruption in BBB integrity allows large molecules, even living cells, infiltrate into brain parenchyma. Systemic autoimmune diseases in lupus patients or in autoimmune mouse models, frequently showed comprised BBB [33] and T cells invasion into brain [34]. This is indeed the case for TKO mice. T cells can be easily detected in all TKO brains we examined (n = 10), since the infiltrating cells in the TKO brains displayed immunoreactivity to CD3, indicating the identity of the T lymphocytes (Fig. 4B). The scattered CD3-positive cells were presented within entire TKO brain and high density of those CD3-reactive cells was found within the choroid plexus of the mutant mice (data not shown). These CD3-positive cells were not readily present in the age-matched WT controls (Fig. 4A). In the old mice, lymphocyte infiltration became more noticeable, in some extreme cases (two of the ten mice examined), the cells form colonies in the meningeal area (Fig. 4C), and gradually invaded into cortex along the blood microvessels (Fig. 4C, arrows). T cell infiltration into the TKO brains was further confirmed with flow cytometric analysis of TCRαβ population in the total brain mononuclear cells prepared from the interphase of the 30%/70% Percoll gradient centrifugation of the brain single cell suspension. This again demonstrated that TKO brain retained more than 7 times of TCRαβ positive cells as compared to the WT control (Fig. 4E and 4F, 5.6% vs. 0.8%).

**Figure 5:**
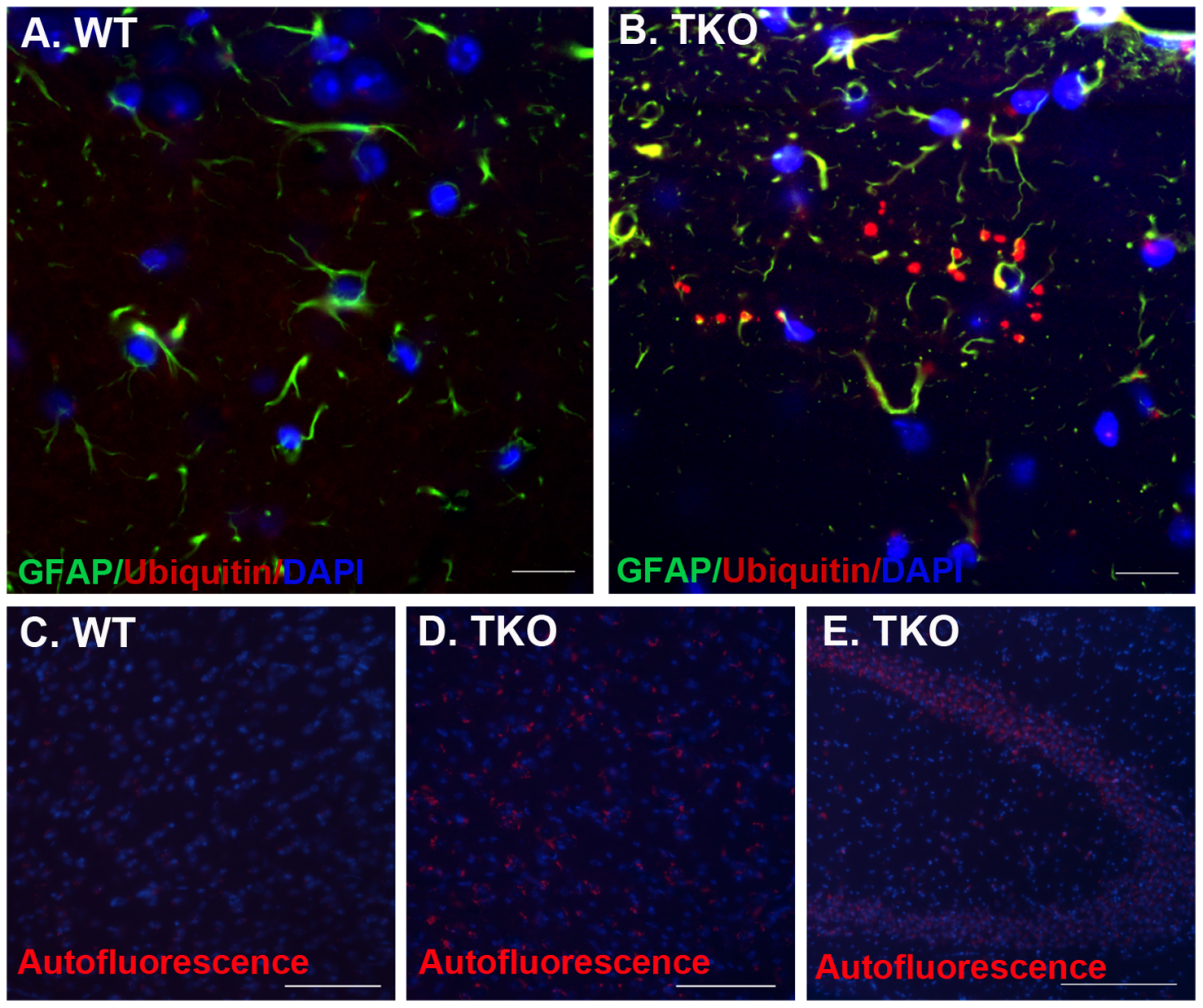
TKO brains develop ubiquitinated protein-aggregates and lipofuscin deposition. (A, B) Ubiquitinated protein aggregates were detected by anti-ubiquitin antibody. The brain cryosections from TKO (n = 5) or WT (n = 3) mice at ages of 6–8 months were prepared as described in Methods and Materials. 20 micron of cryostat sections was cut and immunostained with anti-ubiquitin (red, 1∶100, RnD) and -GFAP (green, 1∶200, Santa Cruz) antibodies. Nuclei were stained blue with DAPI. Fluorescent images were obtained with a confocal microscope (LSM510, Zeiss). (C–E) Lipofuscin autofluorescence was accumulated in the TKO brains. The brain sections were prepared from the WT (C) and TKO (D and E) mice at age of 2 months old, and stained with DAPI for cell nuclei; the sections were observed and photographed under UV laser light. The lipofuscins were shown as red aggregates at one side of blue-stained nuclei. Scale Bars are 50 µm in (A and B), and 100 µm in (C–E).

### Ubiquitinated Protein Inclusions are Accumulated in the TKO Brains

Insulting attack from autoreactive inflammation in the TKO brain led us to investigate its effect on neuronal physiology. Ubiquitination is a tagging process for malfunctional or spent proteins that were targeted for proteasomal degradation. Inefficacy in removal of those ubiquitinated proteins and intracellular accumulation of toxic metabolites has been proposed to contribute to cell death in the aged brains [35]–[37]. We, therefore, examined the ubiquitination in TKO brains and found that ubiquitin-immunopositive dot-like structures were present in the mutant brains, particularly within the strata oriens, pyramidale and radiatum of the CA3 region of the hippocampus (Fig. 5B). Count on ubiquitin-positive dots was performed on coronal sections at same level across hippocampus in five TKO and three WT brains, and showed significant difference with average of 5 clusters of ubiquitin-aggregates in each section across the TKO hippocampus, but zero in those of WT brains (Fig. 5A). In addition, lipofuscin formation, as revealed by the presence of auto-fluorescent aggregates under UV microscope, was accumulated around nuclei and the autofluorescent intensity was dramatically increased in the TKO brains (Fig. 5D and 5E) than in the WT brains (Fig. 5C), suggesting a defective or inefficient breakdown of lipoprotein by the TKO cells.

### Timm Staining Reveals Hippocampal Hilar Mossy Fiber Damages in TKO Mice

**Figure 6:**
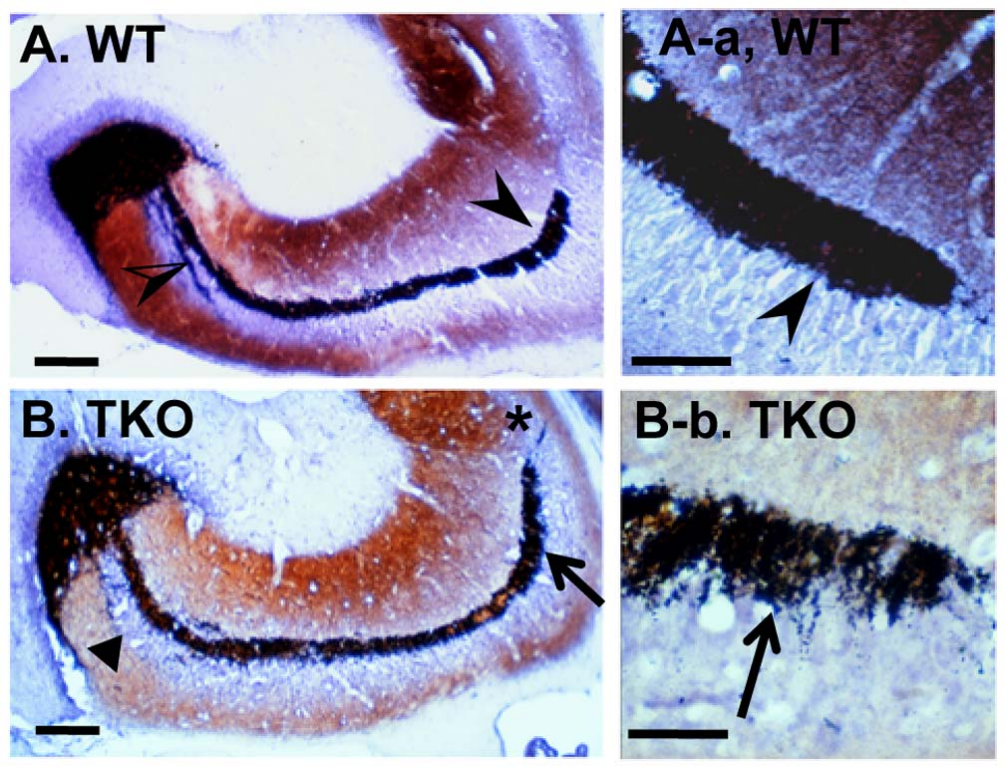
The TKO hippocampal CA3 regions show altered mossy fiber projection and sprouting. Timm staining of the Zn^2+^-containing mossy fiber terminals was performed on the hippocampus of WT and TKO mice at ages of 8–10 months. (A and A-a) Mossy fibers in the WT brains were terminated in the stratum lucidum, above the CA3 pyramidal cells layer (arrowheads), and below and within the pyramidal cell layer in the proximal portion of CA3 (half-open arrowhead). (B and B-b) Mossy fiber sprouting in the suprapyramidal bundle (arrows), and a fuzzy distal boundary of the suprapyramidal bundle spreading across the pyramidal cell layer into CA2 (asterisk) were shown on the TKO brain sections. Triangle in (B) shows the lightly stained and diffused intra and infrapyramidal bundles. Scale bars, 100 µm in (A and B), and 50 µm in (A-a and B-b).

Accumulation of ubiquitinated protein aggregates and autofluorescent lipofuscins in the TKO hippocampus prompted us to further study hippocampal morphological changes by examination of the integrity of mossy fiber that projects from dentate granule cells to the stratum lucidum layer of pyramidal cells in the CA3 region. Timm staining is a sulfide-silver method that detects zinc with an insoluble sulfide, and is commonly used to examine mossy fiber sprouting resulting from degenerating axon terminals, for most time, caused by seizure, and by other insulting damages as well [38]. Timm reactive mossy fibers in the WT brains terminated in a narrow zone of the stratum lucidum, just above the CA3 pyramidal cells layer (Fig. 6A and 6A-a arrowheads), and they also terminated below and within the pyramidal cell layer in the proximal portion of CA3 (Fig. 6A, half-open arrowhead). However, such narrow mossy fiber projection with clear boundary seen in the WT brains was disturbed in the TKO brains prepared from the TKO mice at ages of 8–10 months, as characterized with mossy fiber sprouting occurring in the suprapyramidal bundle (Fig. 6B and 6B-b, arrows) and a fuzzy distal boundary of the suprapyramidal bundle spreading across the pyramidal cell layer into CA2 (Fig. 6B, asterisk). The intra and infrapyramidal bundles were lightly stained and diffused (Fig. 6B, triangle). These changes are likely as a consequence of dentate granule cell damage, since those cells have been shown as most vulnerable and among the first neurons to die in response to excitotoxic insults [39], [40]. Such mossy fiber-sprouting pattern was never seen in WT controls (Fig. 6A).

### TUNEL Positive Apoptotic Cells were Detected in TKO Brain Including Dentate Gyrus

**Figure 7:**
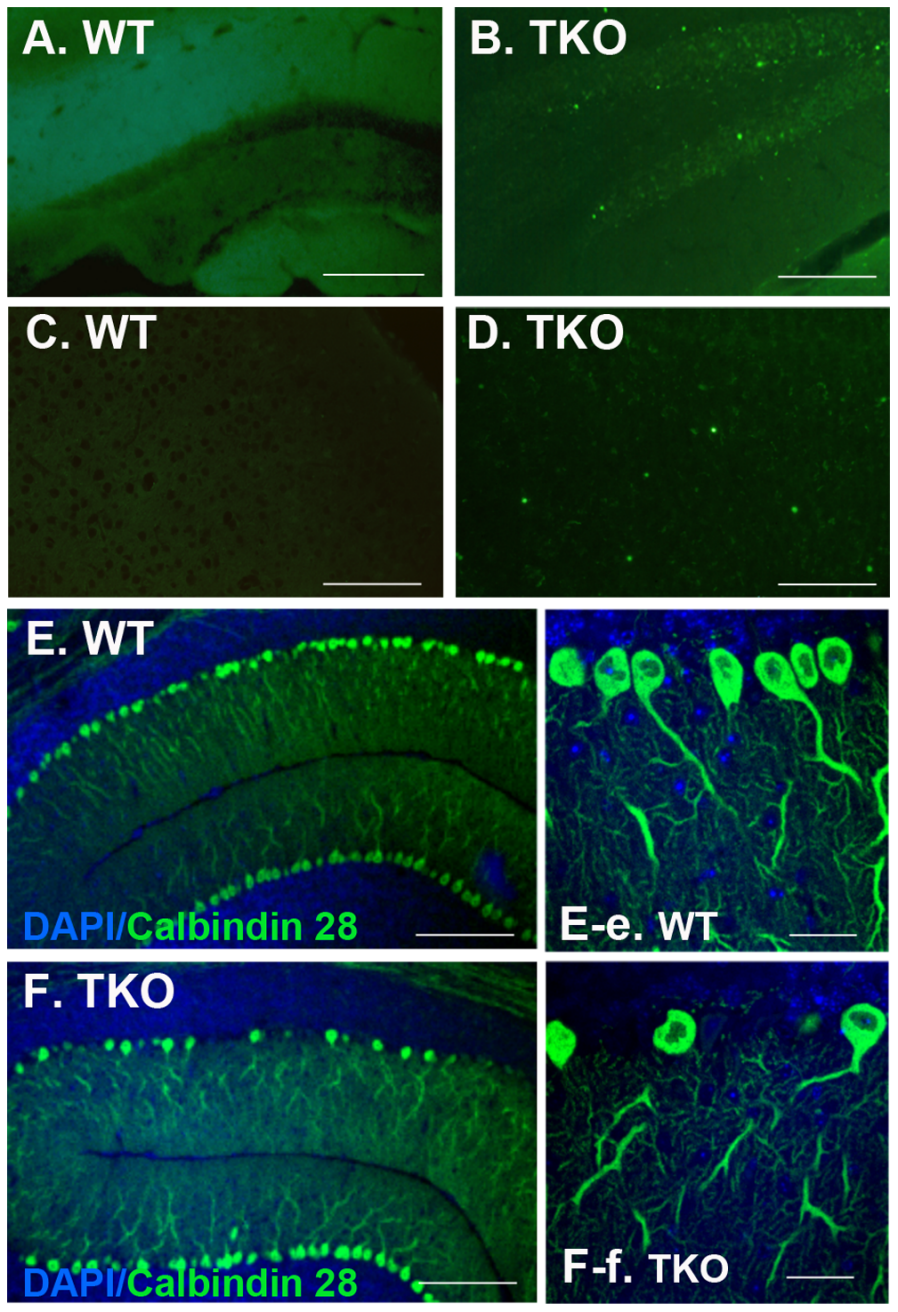
TKO brains show increased apoptosis and cell death. Mice at 6–8 weeks of age were deeply anesthetized by 2.5% Avertin prior to transcardial perfusion with 1 unit/mL of heparin dissolved in 1×PBS. Brains were harvested, cryosectioned at 20 micron. (A–D) TUNEL labeling showed increased apoptosis in the TKO brains (B and D) as compared to the negative labeling in the WT brains (A and C). (E and F) Purkinje cell loss was shown in the TKO cerebellum (F and F-f) but not in the WT brains (E and E-e). Purkinje cells were immunostained with antibody for calbindin D28K and cell nuclei were stained with DAPI. Fluorescent images were obtained with a confocal microscope (LSM510, Zeiss). Scale Bars in (A–F), 100 µm; and in (E-e and F-f), 50 µm.

Formation of ubiquitinated protein inclusions and disorganization of mossy fibers imply a neuronal injury in the TKO brains. We therefore performed TUNEL assay on brain sections from 6 WT and 6 TKO mice at ages of 8–10 months in two independent experiments, and showed that the TUNEL-positive apoptotic cells were clearly present in a variety of brain regions, most affected regions were hippocampal dentate gyrus (Fig. 7B); and others included cortex with scattering TUNEL labeling in the lays III–V of cortical area (Fig. 7D); thalamus and hypothalamus; corpus callosum; anterior alfactory nucleus; both sides of the rostral nucleus accumbens; paraolivary nucleus and superior olivary nucleus; and brainstem particularly in the Nucleus Raphe Obscurus and the nucleus Roller; and external granular layer of the cerebellum. Meanwhile, the TUNEL assay showed negligible labeling in the age-matched WT controls (Fig. 7A and 7C). Although the TUNEL labeling was not readily seen in the Purkinje layer of TKO cerebellum, degeneration in Purkinje cells were clearly present (Fig. 7F and 7F-f), as shown by sporadic loss of calbindin-28 positive cells in the Purkinje layer and such cell loss was increased with aging. This is in consistent with phenotype found in the mouse models of experimental autoimmune encephoalomyelitis (EAE), in which the Purkinje cells were highly vulnerable to autoimmune reactivity [41]. Our data further support the observation that the autoimmune MRL-lpr mice and SLE patients show neuronal loss in the CA3 region and dentate gyrus regions [42], [43].

## Discussion

TAM receptor knockout mice develop spontaneous lupus-like autoimmune diseases [1] with characteristics of splenomegaly, autoantibodies deposition in glomeruli and lymphocyte infiltration into tissues including retina [7]. We further demonstrated, in the present study, that the different subclasses of immunoglobulin level were dramatically increased in the mutant mice with isotype switch favoring autoimmune disorders; and the autoreactive antibodies were clearly deposited along the inner wall of the brain blood vessels. Although such accumulation of antibodies along the blood vessel is not very commonly noticed in the CNS autoimmune disease models, autoantibodies against a variety of CNS components including myelin basic protein, myelin-associated glycoprotein, neuronal cell-surface, -intracellular molecules, neuronal transmitter receptors, have been found to participate in demyelination and disease progression in the multiple sclerosis patients [44]–[47].

Systemic autoimmune disorders usually produce increased level of pro-inflammatory cytokines which are able to drive peripheral autoimmunity into the CNS. A higher level of circulating TNF-α was found in TKO blood, which may cause abnormal activation of endothelial cells [1]. TNF-α increases BBB permeability and induces intracellular adhesion molecule (ICAM) expression on endothelial cells [48], [49]. Such cytokine-driven and exaggerated ICAM-dependent leukocyte-endothelial interactions were also found in the brain of MRL–lpr mice [50], a mouse model systemic autoimmune disease that spontaneously develops manifestation of human multiple sclerosis disease. In agreement with this, we also demonstrated that CD3 positive T cells were infiltrated into parenchymal brain tissue, causing neuroinflammation in TKO brains. T-cell mediated inflammation has been considered as major factor causing demyelination and damage to oligodendrocytes in the multiple sclerosis disorders.

Systemic autoimmunity or CNS-specific autoimmune diseases, such as EAE in mouse or multiple sclerosis in human, impair BBB permeability. Similarly, the TKO brains exhibit age-related increase in BBB permeability as shown by increased perivascular leakage of Evans Blue and FITC-dextran around brain vessels. Such increased BBB permeability might be caused by autoimmune disorders in which the autoantibodies reactive to endothelial antigens or the higher level of proinflammatory cytokines might target on brain vessels, causing brain damages. However, it is worthy to note, all three members of the TAM receptors are expressed by endothelial cells [11], [14]; and the protein S knockout causes embryonic lethality due to incomplete development of vascular system, BBB disruption and intracerebral hemorrhages [12], [13]. Axl and its ligand, Gas6, have been shown to promote smooth muscle and endothelial cells survival, migration [14]. Tyro3 is critical in supporting endothelial cell adhesion and BBB integrity in an in vitro hypoxic assay system or during transient middle cerebral artery occlusion procedure [11]. Protein S protects post-ischemic BBB disruption in *Tyro3^+/+^Axl^−/−^Mertk^−/−^* but not *Tyro3^−/−^* mice, suggesting Protein S and Tyro3 signaling pathway plays important role in supporting BBB integrity [11]. Notably, the TAM receptors play redundant roles in many cases [1], [10], we therefore tested BBB permeability in the TKO. Although we did not observed obvious perivascular leakage in the naïve *Tyro3^−/−^* mice without any ischemic induction, we indeed found leakage of Evans blue and fluorescein-dextran into brain parenchyma in the TKO mice and the permeability of the cultured brain endothelial monolayer prepared from either TKO or both *Axl^−/−^Mertk^−/−^* and *Tyro3^−/−^Axl^−/−^* double knockout mice was dramatically increased. Those data suggest that lacking of TAM expression on the blood vessels and systemic autoimmune disorders in the TAM TKO mice may collectively contribute to TAM TKO BBB disruption, and the autoimmunity probably played more determinant role in causing BBB permeability in those untreated TKO mice. Our data further support previous observation that the MRL mouse models and lupus patients exhibited increased BBB permeability and blood vessel disruption [43].

Autoimmune insulting and breakdown of BBB protection endanger brain cell integrity and neuronal functions. Close examination of the brain damages in the TKO mice clearly showed difference from those found in the EAE models and the MS patients, in which the autoreactive T cell or autoantibodies attacked myelin causing demyelination and neurodegeneration. Premature lipofuscin deposition was evident in the TKO neurons within entire brain, and ubiquitinated aggregates developed in the hippocampal complex; and most likely those ubiquitinated aggregates represent the degenerating axon terminals. These results suggest that autoimmune mediated neuroinflammation can impair ubiquitin-dependent proteolysis, which may count, in a large part, for hippocampal damage and neuronal cell death as observed in the TKO mice. Such defective ubiquitin-dependent proteolysis has been considered to contribute largely for neuronal death in many chronic neurodegenerative diseases [51], [52], including Alzheimer’s disease, Parkinson’s disease, Amyotrophic Lateral Sclerosis and Hintington’s disease [52], [53]. Inefficient elimination of these ubiquitinated proteins leads to neurodegeneration [24], [52], [54]. We constantly detected TUNEL-positive apoptotic cell death in a variety of brain regions, particularly in hippocampus, consistent with the presence of ubiquitinated aggregates found predominantly in TKO hippocampal formation. It is conceivable that emergence of autoimmunity in TKO brain alters the ubiquitin-proteasome degradation system, which in turn causes apoptotic neuronal cell death in the mutant brain.

Hippocampus has been shown to be the most vulnerable to chronic inflammation, and the structural alterations are present in TKO hippocampus as revealed by Timm staining that is commonly used to show the integrity of mossy fibers. Inflammatory response within CNS modulates homeostasis of newborn cells in the adult dentate gyrus by inhibition of neuronal stem cell proliferation and causing mature neuron death, which may interrupt proper axonal targeting of mossy fibers in TKO hippocampus. In chronic autoimmune diseases of CNS, such as multiple sclerosis, the clinical signs of cognitive dysfunction, such as deficits in attention, information processing, and long-term memory have been associated with structural changes in the hippocampus [55]. Since all three members of TAM receptors are expressed in neuron with predominant expression of Tyro3, functional impairment may be caused by direct loss of TAM receptors. However, the structural changes and functional deteriorations in TKO mice took place gradually with aging and the progression of systemic autoimmunity, suggesting that autoimmune disorders contribute significantly to the hippocampal atrophy in those TKO mice. Our observation on the TAM TKO mice demonstrates that systemic autoimmunity and neuroinflammation contribute significantly to hippocampal damage and neuronal loss. This knockout model, in many aspects, represents manifestations of systemic autoimmunity in SLE patients or the animal models of lupus, as well as the multiple sclerosis, which display atrophy of pyramidal neurons [56], deficits in spatial learning/memory tasks [57], and impaired cognitive functions [58].

## References

[1] Lu Q, Lemke G (2001) Homeostatic regulation of the immune system by receptor tyrosine kinases of the Tyro 3 family. Science 293: 306–311. 11452127 10.1126/science.1061663

[2] Lemke G, Lu Q (2003) Macrophage regulation by Tyro 3 family receptors. Curr Opin Immunol 15: 31–36. 12495730 10.1016/s0952-7915(02)00016-x

[3] Rothlin CV, Ghosh S, Zuniga EI, Oldstone MB, Lemke G (2007) TAM receptors are pleiotropic inhibitors of the innate immune response. Cell 131: 1124–1136. 18083102 10.1016/j.cell.2007.10.034

[4] Wallet MA, Sen P, Flores RR, Wang Y, Yi Z, et al (2008) MerTK is required for apoptotic cell-induced T cell tolerance. J Exp Med 205: 219–232. 18195070 10.1084/jem.20062293 PMC2234377

[5] Radic MZ, Shah K, Zhang W, Lu Q, Lemke G, et al (2006) Heterogeneous nuclear ribonucleoprotein P2 is an autoantibody target in mice deficient for Mer, Axl, and Tyro3 receptor tyrosine kinases. J Immunol 176: 68–74. 16365397 10.4049/jimmunol.176.1.68 PMC1564271

[6] Ye F, Han L, Lu Q, Dong W, Chen Z, et al (2011) Retinal self-antigen induces a predominantly Th1 effector response in Axl and Mertk double-knockout mice. J Immunol 187: 4178–4186. 21918185 10.4049/jimmunol.1101201 PMC3190567

[7] Ye F, Li Q, Ke Y, Lu Q, Han L, et al (2011) TAM receptor knockout mice are susceptible to retinal autoimmune induction. Invest Ophthalmol Vis Sci 52: 4239–4246. 21467176 10.1167/iovs.10-6700 PMC3175940

[8] Prieto AL, Weber JL, Lai C (2000) Expression of the receptor protein-tyrosine kinases Tyro-3, Axl, and mer in the developing rat central nervous system. J Comp Neurol 425: 295–314. 10954847

[9] Prieto AL, O'Dell S, Varnum B, Lai C (2007) Localization and signaling of the receptor protein tyrosine kinase Tyro3 in cortical and hippocampal neurons. Neuroscience 150: 319–334. 17980494 10.1016/j.neuroscience.2007.09.047 PMC2231337

[10] Lu Q, Gore M, Zhang Q, Camenisch T, Boast S, et al (1999) Tyro-3 family receptors are essential regulators of mammalian spermatogenesis. Nature 398: 723–728. 10227296 10.1038/19554

[11] Zhu D, Wang Y, Singh I, Bell RD, Deane R, et al (2010) Protein S controls hypoxic/ischemic blood-brain barrier disruption through the TAM receptor Tyro3 and sphingosine 1-phosphate receptor. Blood 115: 4963–4972. 20348395 10.1182/blood-2010-01-262386 PMC2890172

[12] Burstyn-Cohen T, Heeb MJ, Lemke G (2009) Lack of protein S in mice causes embryonic lethal coagulopathy and vascular dysgenesis. J Clin Invest 119: 2942–2953. 19729839 10.1172/JCI39325 PMC2752078

[13] Saller F, Brisset AC, Tchaikovski SN, Azevedo M, Chrast R, et al (2009) Generation and phenotypic analysis of protein S-deficient mice. Blood 114: 2307–2314. 19567881 10.1182/blood-2009-03-209031 PMC2745849

[14] Melaragno MG, Cavet ME, Yan C, Tai LK, Jin ZG, et al (2004) Gas6 inhibits apoptosis in vascular smooth muscle: role of Axl kinase and Akt. J Mol Cell Cardiol 37: 881–887. 15380678 10.1016/j.yjmcc.2004.06.018

[15] Holland SJ, Powell MJ, Franci C, Chan EW, Friera AM, et al (2005) Multiple roles for the receptor tyrosine kinase axl in tumor formation. Cancer Res 65: 9294–9303. 16230391 10.1158/0008-5472.CAN-05-0993

[16] Korshunov VA, Mohan AM, Georger MA, Berk BC (2006) Axl, a receptor tyrosine kinase, mediates flow-induced vascular remodeling. Circ Res 98: 1446–1452. 16627783 10.1161/01.RES.0000223322.16149.9a

[17] Ozaki H, Ishii K, Horiuchi H, Arai H, Kawamoto T, et al (1999) Cutting edge: combined treatment of TNF-alpha and IFN-gamma causes redistribution of junctional adhesion molecule in human endothelial cells. J Immunol 163: 553–557. 10395639

[18] Dobbie MS, Hurst RD, Klein NJ, Surtees RA (1999) Upregulation of intercellular adhesion molecule-1 expression on human endothelial cells by tumour necrosis factor-alpha in an in vitro model of the blood-brain barrier. Brain Res 830: 330–336. 10366690 10.1016/s0006-8993(99)01436-5

[19] Dickstein JB, Moldofsky H, Hay JB (2000) Brain-blood permeability: TNF-alpha promotes escape of protein tracer from CSF to blood. Am J Physiol Regul Integr Comp Physiol 279: R148–151. 10896876 10.1152/ajpregu.2000.279.1.R148

[20] Tsao N, Hsu HP, Wu CM, Liu CC, Lei HY (2001) Tumour necrosis factor-alpha causes an increase in blood-brain barrier permeability during sepsis. J Med Microbiol 50: 812–821. 11549183 10.1099/0022-1317-50-9-812

[21] Yang GY, Gong C, Qin Z, Liu XH, Lorris Betz A (1999) Tumor necrosis factor alpha expression produces increased blood-brain barrier permeability following temporary focal cerebral ischemia in mice. Brain Res Mol Brain Res 69: 135–143. 10350645 10.1016/s0169-328x(99)00007-8

[22] Jacob A, Hack B, Chiang E, Garcia JG, Quigg RJ, et al (2010) C5a alters blood-brain barrier integrity in experimental lupus. FASEB J 24: 1682–1688. 20065106 10.1096/fj.09-138834 PMC2874478

[23] Ballok DA, Millward JM, Sakic B (2003) Neurodegeneration in autoimmune MRL-lpr mice as revealed by Fluoro Jade B staining. Brain Res 964: 200–210. 12576180 10.1016/s0006-8993(02)03980-x

[24] Sakic B, Maric I, Koeberle PD, Millward JM, Szechtman H, et al (2000) Increased TUNEL staining in brains of autoimmune Fas-deficient mice. J Neuroimmunol 104: 147–154. 10713354 10.1016/s0165-5728(99)00277-5

[25] Hanly JG, Robichaud J, Fisk JD (2006) Anti-NR2 glutamate receptor antibodies and cognitive function in systemic lupus erythematosus. J Rheumatol 33: 1553–1558. 16881112

[26] Suh CH, Hilliard B, Li S, Merrill JT, Cohen PL (2010) TAM receptor ligands in lupus: protein S but not Gas6 levels reflect disease activity in systemic lupus erythematosus. Arthritis Res Ther 12: R146. 20637106 10.1186/ar3088 PMC2945040

[27] Bullard DC, Hu X, Schoeb TR, Collins RG, Beaudet AL, et al (2007) Intercellular adhesion molecule-1 expression is required on multiple cell types for the development of experimental autoimmune encephalomyelitis. J Immunol 178: 851–857. 17202346 10.4049/jimmunol.178.2.851

[28] Smith SS, Ludwig M, Wohler JE, Bullard DC, Szalai AJ, et al (2008) Deletion of both ICAM-1 and C3 enhances severity of experimental autoimmune encephalomyelitis compared to C3-deficient mice. Neurosci Lett 442: 158–160. 18634851 10.1016/j.neulet.2008.07.005 PMC2556246

[29] Zandman-Goddard G, Chapman J, Shoenfeld Y (2007) Autoantibodies involved in neuropsychiatric SLE and antiphospholipid syndrome. Semin Arthritis Rheum 36: 297–315. 17258299 10.1016/j.semarthrit.2006.11.003

[30] Camenisch TD, Koller BH, Earp HS, Matsushima GK (1999) A novel receptor tyrosine kinase, Mer, inhibits TNF-alpha production and lipopolysaccharide-induced endotoxic shock. J Immunol 162: 3498–3503. 10092806

[31] Caraux A, Lu Q, Fernandez N, Riou S, Di Santo JP, et al (2006) Natural killer cell differentiation driven by Tyro3 receptor tyrosine kinases. Nat Immunol 7: 747–754. 16751775 10.1038/ni1353

[32] Sharif MN, Sosic D, Rothlin CV, Kelly E, Lemke G, et al (2006) Twist mediates suppression of inflammation by type I IFNs and Axl. J Exp Med 203: 1891–1901. 16831897 10.1084/jem.20051725 PMC2118370

[33] Abbott NJ, Mendonca LL, Dolman DE (2003) The blood-brain barrier in systemic lupus erythematosus. Lupus 12: 908–915. 14714910 10.1191/0961203303lu501oa

[34] Ma X, Foster J, Sakic B (2006) Distribution and prevalence of leukocyte phenotypes in brains of lupus-prone mice. J Neuroimmunol 179: 26–36. 16904195 10.1016/j.jneuroim.2006.06.023

[35] DeKosky ST, Scheff SW (1990) Synapse loss in frontal cortex biopsies in Alzheimer's disease: correlation with cognitive severity. Ann Neurol 27: 457–464. 2360787 10.1002/ana.410270502

[36] Samuel W, Masliah E, Hill LR, Butters N, Terry R (1994) Hippocampal connectivity and Alzheimer's dementia: effects of synapse loss and tangle frequency in a two-component model. Neurology 44: 2081–2088. 7969963 10.1212/wnl.44.11.2081

[37] Gray DA, Tsirigotis M, Woulfe J (2003) Ubiquitin, proteasomes, and the aging brain. Sci Aging Knowledge Environ 2003: RE6. 12944592 10.1126/sageke.2003.34.re6

[38] Jiao Y, Nadler JV (2007) Stereological analysis of GluR2-immunoreactive hilar neurons in the pilocarpine model of temporal lobe epilepsy: correlation of cell loss with mossy fiber sprouting. Exp Neurol 205: 569–582. 17475251 10.1016/j.expneurol.2007.03.025 PMC1995080

[39] Sloviter RS, Zappone CA, Harvey BD, Bumanglag AV, Bender RA, et al (2003) “Dormant basket cell” hypothesis revisited: relative vulnerabilities of dentate gyrus mossy cells and inhibitory interneurons after hippocampal status epilepticus in the rat. J Comp Neurol 459: 44–76. 12629666 10.1002/cne.10630

[40] Lowenstein DH, Thomas MJ, Smith DH, McIntosh TK (1992) Selective vulnerability of dentate hilar neurons following traumatic brain injury: a potential mechanistic link between head trauma and disorders of the hippocampus. J Neurosci 12: 4846–4853. 1464770 10.1523/JNEUROSCI.12-12-04846.1992 PMC6575779

[41] MacKenzie-Graham A, Tiwari-Woodruff SK, Sharma G, Aguilar C, Vo KT, et al (2009) Purkinje cell loss in experimental autoimmune encephalomyelitis. Neuroimage 48: 637–651. 19589388 10.1016/j.neuroimage.2009.06.073 PMC2754586

[42] Schnider A, Bassetti C, Gutbrod K, Ozdoba C (1995) Very severe amnesia with acute onset after isolated hippocampal damage due to systemic lupus erythematosus. J Neurol Neurosurg Psychiatry 59: 644–646. 10.1136/jnnp.59.6.644-a PMC1073770 7500113

[43] Ballok DA, Woulfe J, Sur M, Cyr M, Sakic B (2004) Hippocampal damage in mouse and human forms of systemic autoimmune disease. Hippocampus 14: 649–661. 15301441 10.1002/hipo.10205 PMC1764443

[44] Vyshkina T, Kalman B (2008) Autoantibodies and neurodegeneration in multiple sclerosis. Lab Invest 88: 796–807. 18521063 10.1038/labinvest.2008.53

[45] Genain CP, Cannella B, Hauser SL, Raine CS (1999) Identification of autoantibodies associated with myelin damage in multiple sclerosis. Nat Med 5: 170–175. 9930864 10.1038/5532

[46] O'Connor KC, Appel H, Bregoli L, Call ME, Catz I, et al (2005) Antibodies from inflamed central nervous system tissue recognize myelin oligodendrocyte glycoprotein. J Immunol 175: 1974–1982. 16034142 10.4049/jimmunol.175.3.1974 PMC4515951

[47] Vaknin-Dembinsky A, Weiner HL (2007) Relationship of immunologic abnormalities and disease stage in multiple sclerosis: implications for therapy. J Neurol Sci 259: 90–94. 17521671 10.1016/j.jns.2006.11.022

[48] Nishioku T, Matsumoto J, Dohgu S, Sumi N, Miyao K, et al (2010) Tumor necrosis factor-alpha mediates the blood-brain barrier dysfunction induced by activated microglia in mouse brain microvascular endothelial cells. J Pharmacol Sci 112: 251–254. 20118615 10.1254/jphs.09292sc

[49] McHale JF, Harari OA, Marshall D, Haskard DO (1999) TNF-alpha and IL-1 sequentially induce endothelial ICAM-1 and VCAM-1 expression in MRL/lpr lupus-prone mice. J Immunol 163: 3993–4000. 10491002

[50] James WG, Hutchinson P, Bullard DC, Hickey MJ (2006) Cerebral leucocyte infiltration in lupus-prone MRL/MpJ-fas lpr mice–roles of intercellular adhesion molecule-1 and P-selectin. Clin Exp Immunol 144: 299–308. 16634804 10.1111/j.1365-2249.2006.03056.x PMC1809650

[51] Li Z, Jansen M, Pierre SR, Figueiredo-Pereira ME (2003) Neurodegeneration: linking ubiquitin/proteasome pathway impairment with inflammation. Int J Biochem Cell Biol 35: 547–552. 12672447 10.1016/s1357-2725(02)00384-9

[52] Ciechanover A, Brundin P (2003) The ubiquitin proteasome system in neurodegenerative diseases: sometimes the chicken, sometimes the egg. Neuron 40: 427–446. 14556719 10.1016/s0896-6273(03)00606-8

[53] Sherman MY, Goldberg AL (2001) Cellular defenses against unfolded proteins: a cell biologist thinks about neurodegenerative diseases. Neuron 29: 15–32. 11182078 10.1016/s0896-6273(01)00177-5

[54] Layfield R, Alban A, Mayer RJ, Lowe J (2001) The ubiquitin protein catabolic disorders. Neuropathol Appl Neurobiol 27: 171–179. 11489136 10.1046/j.1365-2990.2001.00335.x

[55] Chiaravalloti ND, DeLuca J (2008) Cognitive impairment in multiple sclerosis. Lancet Neurol 7: 1139–1151. 19007738 10.1016/S1474-4422(08)70259-X

[56] Sakic B, Szechtman H, Denburg JA, Gorny G, Kolb B, et al (1998) Progressive atrophy of pyramidal neuron dendrites in autoimmune MRL-lpr mice. J Neuroimmunol 87: 162–170. 9670858 10.1016/s0165-5728(98)00085-x

[57] Sakic B, Szechtman H, Denburg S, Carbotte R, Denburg JA (1993) Spatial learning during the course of autoimmune disease in MRL mice. Behav Brain Res 54: 57–66. 8504012 10.1016/0166-4328(93)90048-u

[58] Tomietto P, Annese V, D'Agostini S, Venturini P, La Torre G, et al (2007) General and specific factors associated with severity of cognitive impairment in systemic lupus erythematosus. Arthritis Rheum 57: 1461–1472. 18050188 10.1002/art.23098

